# Automated Drone‐Delivery Solar‐Driven Onsite Wastewater Smart Monitoring and Treatment System

**DOI:** 10.1002/advs.202302935

**Published:** 2023-06-26

**Authors:** Fengjie He, Ming Zhu, Jiawei Fan, Edwin Ma, Shengjie Zhai, Hui Zhao

**Affiliations:** ^1^ Department of Mechanical Engineering University of Nevada Las Vegas NV 89154 USA; ^2^ Department of Electrical and Computer Engineering Engineering University of Nevada Las Vegas NV 89154 USA; ^3^ Ed W. Clark High School Las Vegas NV 89102 USA

**Keywords:** autonomous analysis, drone delivery, onsite treatment, remote monitoring, silk fibroin bioadsorbent

## Abstract

Treating potential polluted water sources is urgent and challenging, especially for natural water sources. Numerous research groups focus on either smart water monitoring or new adsorbent. However, either aspect alone is insufficient for complex nature water source treatment. Here, integrating the state‐of‐art machine learning technique, a sustainable silk‐based bioadsorbent, and wireless Internet of Things, an integrated automated drone‐delivery solar driven onsite water monitoring & treatment system (WMTS) for the contaminated nature water sources is developed. In short, the embedded sensors and microprogrammed control unit capture and upload the real‐time monitoring data to the cloud server for data analysis and optimized treatment strategy. Meanwhile, a grid map system based on the satellite remote sensing images directs the minimum number of WMTS units to cover the entire polluted region. Finally, unmanned aerial vehicles provide autonomous dispatch, operation, and maintenance, especially in hard‐to‐reach sites. Overall, this work offers a general, sustainable, energy‐efficient, and closed‐loop solution toward efficiently alerting and on‐site treating nature water source contamination.

## Graphical abstract

An integrated automated air‐delivery solar driven onsite water monitoring & treatment system for efficient and sustainable treatment of natural water sources is developed. The system consists of the Internet of Things‐based real‐time and continuous water monitoring unit and the bioadsorbent silk fibroin film‐based sustainable water purification unit. The dispatch and maintenance of the system is carried out by unmanned aerial vehicles.

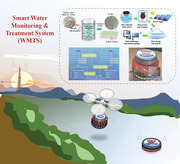

## 1 Introduction

As our personal drinking water sources become increasing contaminated by surrounding environment or human activities, over 780 million people on earth live without access to clean and safe water and every 2 min a child dies from a water‐related disease.^[^ ^1^, ^2^, ^3^, ^4^, ^5^, ^6^, ^7^, ^8^ ^]^ Particularly, in the arid region, there exists severe water shortage due to the climate change and water is a precious resource. Therefore, treating polluted water sources like rivers, lakes, and streams becomes a matter of great urgency to alleviate water scarcity and supply safe drinking water.^[^ ^9^, ^10^ ^]^ However, in general, treating polluted natural water is difficult, costly, and time‐consuming, as natural water bodies are large, dispersed (numerous tributaries), and in dynamic state (due to heavy rain, storm overflows, etc.).^[^ ^11^, ^12^, ^13^, ^14^ ^]^ Furthermore, the types and concentration of the contaminants originating from both point and nonpoint sources into these water bodies differ greatly, complicating the situation. Up to date, the research has mainly focused on either the water quality monitoring^[^ ^15^, ^16^, ^17^, ^18^, ^19^, ^20^, ^21^, ^22^, ^23^ ^]^ or developing materials to purify water,^[^ ^24^, ^25^, ^26^, ^27^, ^28^, ^29^ ^]^ whereas we argue that either aspect alone is insufficient to treat natural water bodies. Additionally, existing monitoring methods for natural water bodies suffer from the long‐term stability (e.g., unpredicted power loss of battery) and most of current water purification technologies are not sustainable (e.g., high energy consumption, negative carbon footprint, and toxic sludge production),^[^ ^30^, ^31^ ^]^ and are unsafe to operate and maintain (especially in the hard‐to‐reach sites).^[^ ^32^ ^]^

To tackle the aforementioned challenges, we design an integrated automated drone‐delivery solar driven onsite water monitoring & treatment system (WMTS). The WMTS consists of the Internet of Things (IoT) and machine learning (ML)‐based real‐time water quality monitoring unit and the sustainable bioadsorbent‐based sludge‐free wastewater purification unit. For the monitoring unit, a set of sensors, namely, total dissolved solids (TDS) sensor, potential of hydrogen (pH) sensor, and dissolved oxygen (DO) sensor, are deployed to measure the real‐time water quality. A solar‐power driven Raspberry Pi (RPi), which is a compact and low power consuming data processor for the long‐term and continuous water quality monitoring, collected the measured data and then uploaded them to the Google Cloud via wireless communication. Simultaneously, the cloud server automatically employs a support vector machine (SVM)‐based ML algorithm to analyze the current and historical data to assess current water quality, upon which one can predict the possible water quality trend over time and design an appropriate purification strategy accordingly for the optimal treatment. For the purification unit, green and sustainable silk fibroin (SF) films are used as the efficient wide‐spectrum bioadsorbent^[^ ^33^, ^34^ ^]^ and they are hosted in a customized 3D‐printed carrier enclosure for higher adsorption capacity. Furthermore, the usage of SF films overcomes the toxic sludge and negative environmental footprint.^[^ ^35^ ^]^ Inspired by the site‐specific treatment of the commonly used decentralized/onsite wastewater treatment,^[^ ^36^ ^]^ we use a division method mainly based on the pollution level, water body dimension, and the adsorption capacity of adsorbents to grid the polluted natural water body to achieve a better treatment efficiency with a minimum number of WMTS units. Recently, unmanned aerial vehicles (UAVs) have drawn extensive interests in different areas due to their low cost and versatile functionalities, and they have been employed in various applications such as general delivery and wireless communications.^[^ ^37^, ^38^, ^39^, ^40^ ^]^ Herein, UAVs are utilized to autonomously and precisely dispatch all the WMTS units to the corresponding grid sites in a fast, flexible, and safe way based on the GPS coordinates of the mapped grid. To facilitate the practical application, each individual WMTS is integrated into a portable and readily scalable unit by taking advantage of the 3D‐printing technology. In summary, the designed integrated water monitoring and treatment system with the automated operation and little maintenance provides a promising path toward the high‐performance remediation and management of polluted natural water sources.

## 2 Results and Discussion

### 2.1 WMTS Design and Assembly

**Figure 1:**
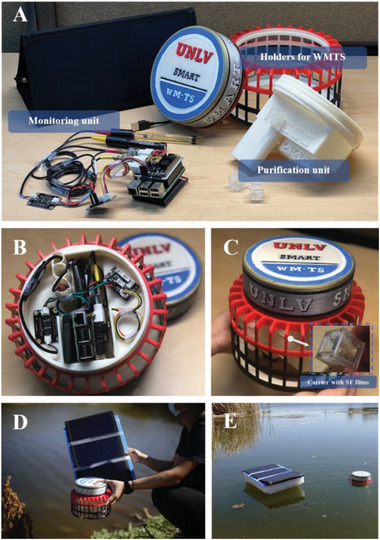
The water monitoring & treatment system (WMTS). A) All components in the WMTS and the customized 3D‐printed holder. B) The monitoring unit inside the holder, C) the assembled WMTS, inset: the SF films inside the 3D‐printed carrier cage. D,E) The assembled WMTS deployed into a lake for water monitoring.

Aiming for adaptive water treatment, each WMTS unit consists of two functional units: a real‐time water‐quality monitoring unit and a sustainable water purification unit (**Figure** **1**A). To enable the reliability and durability of the monitoring unit, a central holder and a cover were designed and fabricated by the 3D printer to encapsulate the hardware away from water (Figure 1B). Meanwhile, the lower part of the central holder was designed in such a way that the sensor probes go through the holder and immerse into the water to measure the water quality. For the water purification unit, the customized 3D‐printed carrier cages with uniform spaces host the SF films (Figure 1C), which enable water to circulate through the SF films and improve the adsorption performance (the technical details will be discussed later). In addition, an outer net holder is used to prevent the cages from being washed away by the occasional strong water flow as well as to enhance the buoyancy of the WMTS in the natural water bodies. The assembled UFO‐like WMTS has a geometry of 21 cm in diameter and 16 cm in height and it can float on the natural water source without any buoy (Figure 1D,E).

### 2.2 The IoT and ML‐Based Water‐Quality Monitoring

**Figure 2:**
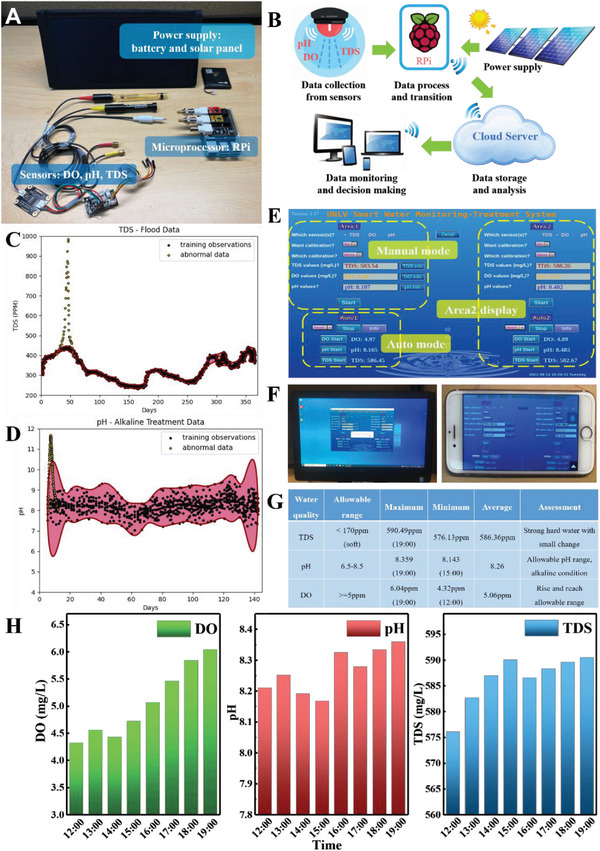
The water quality monitoring system of the WMTS. A) The components of the IoT enabled water monitoring unit. B) The block diagram of the monitoring unit. C) The testing results of the well‐trained SVM models for the TDS indices. D) The testing results of the well‐trained SVM models for the pH indices. E) The UI of the water monitoring and treatment system. F) The UI implemented in the desktop and mobile phone. G) The analysis of the recorded DO, pH, and TDS data from the Railroad Lake. H) Profiles of the DO, pH, and TDS of the Railroad Lake (from left to right).

The designed IoT‐based water‐quality monitoring unit in the WMTS consists of four components: the sensing and data collection unit, microprocessor unit, solar‐based power supply unit, and the cloud‐based data remote monitoring and analysis server (**Figure** **2**A). Wastewater is generally characterized by the considerable amount of weakly biodegradable dissolved contaminants, highly fluctuating pH (from 1 to 14) as well as a relatively low DO (less than 4 ppm).^[^ ^41^, ^42^, ^43^ ^]^ Herein, we examined the TDS, pH, and DO values as the indices of water quality by immerging the related sensor probes into water. A compact RPi was employed as the core microprocessor to continuously gather and monitor these water quality data and upload them to the remote Google cloud server via the built‐in Wi‐Fi communication model (see the diagram in Figure 2B).

The cloud server is equipped with an ML model (i.e., one‐class SVM) that is pre‐trained with both synthetic and practical water quality data to learn the “normal” water quality pattern. The WMTS unit keeps sending real‐time water quality data to the cloud server, and the server classifies the data into either “normal” or “abnormal.” For the training outcomes of the SVM models for TDS and pH indices (see Figure S1, Supporting Information), the black dots represent the historical water quality data that are used for training, and the pink region represents the “normal” data range over time. Here, the DO is not used for the water quality detection, as it is determined by the metabolism of the organisms living within the body of water and cannot respond effectively and swiftly for most abnormalities. Once the model is trained, it can be deployed to examine new data (i.e., testing) and determine if they are “normal.” As shown in Figure 2C,D, the black dots within the pink region are determined as “normal,” then no action is necessary. Otherwise, if the new data dots are outside of the pink region (probably due to the flooding or other occasions), it is determined as “abnormal,” the system will push notifications and countermeasures to the operators (see the detailed flowchart in Figure S2, Supporting Information). Moreover, the future normal range of water quality can also be predicted based on the historical data, thereby prevention treatment can be applied in advance. To make it user‐friendly, a customized Graphic User Interface (GUI) is designed to display the measured data and implement simple commands like sensor calibration (Figure 2E). Two operational modes are designed for data recording and display: automatic mode and manual mode. Both modes can continuously upload data to the cloud and update them on remote App or GUI for long‐term use. However, under the automatic mode, the latest water‐quality data from sensors are automatically recorded with a fixed preset frequency (i.e., 2s) for real‐time updates and display on the GUI. Thus, the automatic mode is preferable for the adaptive wastewater treatment, in which the real‐time water quality analysis is necessary to deploy a rapid response. On the contrary, for the manual mode, the water quality data recording frequency needs to be preset manually before use, such as every hour, every day, every week, or every month. This mode generally is suitable for monitoring stable water sources such as lakes and ponds with slight water quality change for lower energy consumption. Moreover, the GUI of the monitoring unit is implemented for cross‐platforms (like desktops and mobile phones) in an open access mobile‐web via the Wi‐Fi communication (Figure 2F), and the data saved as .csv files in the cloud can be retrieved for remotely sharing or checking. Here, we used Wi‐Fi for communication. In future, we can also use wireless cellular protocols as an alternative, particularly in areas with good cellular network coverage for long distance monitoring. To be noticed, the RPi is powered by a 12 000 mAh lithium battery, which uses the 22 W solar panel as a recharging source to serve as an uninterruptible power supply (UPS) for the long‐term and continuous work.

To prove the concept, we deployed our monitoring unit for water quality to a 5 m × 5 m water area in the Railroad Lake at Las Vegas Metropolitan area from 12 pm to 7 pm on 19 September 2021. The system could remotely monitor water quality in real time without power interruption. The water quality indices were automatically stored in Google cloud and displayed in the UI of the remote PC. The ML model kept reading and assessing the real‐time water quality data synchronized from the WMTS (Figure 2G). Moreover, the saved .csv file in the cloud could be readily retrieved remotely (Figure 2H). Our observation is that the water quality was relatively stable during the monitoring period, except for the DO values. The DO value of this area increased around 40% from noon (12 pm) to the evening (7 pm), maybe due to the influence of strong sunshine. The pH and TDS only slightly changed, less than 3%. In terms of water quality, both the pH and DO values were within the allowable range for soft water. In contrast, TDS value was out of the allowable range, maybe due to the extra dissolved contaminates like salts, heavy metals, and organic matter in the lake. In addition, to demonstrate that our WMTS is capable of working at different weather conditions, we tested it in a cold windy and rainy day on 25 February, 2023 (Figure S3, Supporting Information). Compared to Figure 2H, the DO values are higher, attributed to the low water temperature as the cold water retains more dissolved oxygen than the warm water.

### 2.3 The SF‐Based Wastewater Purification

Here, the performance of the wastewater purification unit in the WMTS is assessed against textile dyeing industrial wastewater, which is one major wastewater source.^[^ ^44^, ^45^ ^]^ Previous studies demonstrated that SF is capable of interacting with a wide spectrum of water contaminants including all types of water‐soluble synthetic dyes due to the inherent protein components (amino acids), hydrophilic nature, and amphoteric properties.^[^ ^46^, ^47^, ^48^ ^]^

**Figure 3:**
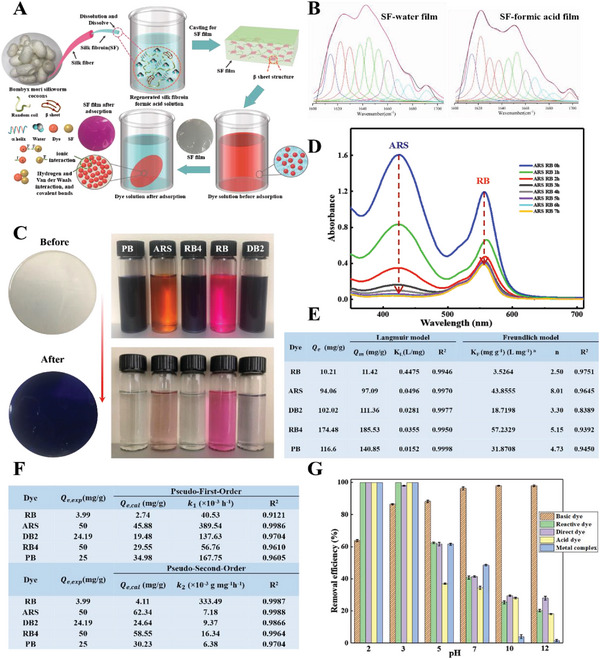
SF film for soluble synthetic dye removal. A) The schematic illustration of the fabrication of SF films and dye removal by the SF films. B) The deconvoluted Fourier transform infrared‐attenuated total reflectance spectra for SF water‐based films and SF formic acid‐based films. C) Photographs of the silk film (for PB) and dye solutions before and after the adsorption by SF films. D) UV‐vis spectra of a mixed dye solution containing ARS (100 ppm) and RB (10 ppm) purified by the SF films as a function of time. E) The parameters in the Langmuir and Freundlich isotherm model fitted for dyes adsorption onto the SF films. F) The parameters in the pseudo‐first‐order and pseudo‐second‐order kinetic model fitted for dye adsorption on the SF film. G) The effects of the dye solution pH value on the removal efficiency of dyes. Conditions: basic dye: RB 10 ppm, anionic dyes: reactive dye RB4 100 ppm, direct dye DB2 40 ppm, acid dye ARS 200 ppm, and metal complex dye PB 100 ppm.

Hence, we use the SF thin films as the bioadsorbent for the dye removal from wastewater (**Figure** **3**A). The bioadsorbent films can be removed from the wastewater without generating toxic sludge. Here, we add formic acid into the conventional SF film self‐assembly process to promote the formation of the crystalline *β*‐sheet in the secondary structure of the SF film (Figure 3B). As a result, the *β*‐sheet content of the SF formic acid‐based film is about 42.2% of the whole secondary structure, whereas the *β*‐sheet content of SF water‐based film is only 27.5% (see details in Table S1, Supporting Information).^[^ ^49^, ^50^ ^]^ The high *β*‐sheet crystalline content has a better resistance to acid and base, making them applicable for the pH value ranging from 1 to 13. This wide applicability in terms of the pH value fits nicely for purifying textile wastewater where pH varies from 2 to 12.^[^ ^51^, ^52^ ^]^ Moreover, the SF films treated with formic acid are shown to be stable in water for several years, preventing secondary pollution from possible degradation of SF films.^[^ ^53^, ^54^ ^]^

To assess the dye removal performance by our fabricated SF films, five commonly used water‐soluble synthetic dyes including Rhodamine B (RB) from basic dyes, Alizarin Red S (ARS) from acid dyes, Direct blue 2 (DB2) from direct dyes, Reactive blue 4 (RB4) from reactive dyes, and Pricion blue H‐5R (PB) from metal complex dyes were chosen as example species. Figure 3C clearly suggests that SF bioabsorbent films are capable of removing various types of dyes from the water with different capacities. The different adsorption capacities for different types of dyes are attributed to different inherent characteristics of dye molecules (i.e., molecular size, ionic nature, and molecular structure).

In addition, we also used the SF films to remove a mixture of dyes including ARS (anionic dye) and RB (cationic dye) (Figure 3D). The UV‐vis spectra of the mixed solution after purification by SF films shows that the intensity of both bands at 425 nm (ARS) and at 554 nm (RB) rapidly decreased in the first hour and then gradually decreased with time, suggesting that our designed SF films can effectively absorb both dyes in the mixture and indicating the broader applicability of our SF films.

Wastewater typically is chelated with heavy metal ions, like mercury (Hg), and chromium (Cr), which worsen the toxicity and complicate the wastewater treatment.^[^ ^55^ ^]^ Here, we examined the adsorption performance of SF films on Hg^2+^ and Cr^6+^. The results demonstrate that the SF film (1.25 cm × 1.25 cm) is capable of removing Hg^2+^ (2.4 ppm) and Cr^6+^ (2.4 ppm) with the efficiency around 86% and 65%, respectively. The capability of removing heavy metal ions further suggests that the SF films as a sustainable bioabsorbent have the potential to treat complicated wastewater.

Next, we calculated the isothermal adsorption coefficient of SF films by examining the adsorption performance of dye solutions with various initial concentrations. As summarized in Figure 3E, the Langmuir model can accurately describe the adsorption process for five different dyes (*R* ^2^ > 0.99) (see details in Figure S4A–E, Supporting Information), suggesting that homogenous monolayer adsorption occurs at binding sites of the SF films.^[^ ^56^, ^57^ ^]^ Furthermore, based on the Langmuir model, we can estimate the maximum theoretical adsorption capacity *Q_m_* of the SF film: 185.53 mg g^−1^ for reactive dye RB4, 97.09 mg g^−1^ for ARS, 111.36 mg g^−1^ for DB2, 140.85 mg g^−1^ for PB, and 11.42 mg g^−1^ for RB. The SF films have a higher adsorption capacity for anionic dyes than that for cationic dyes. It can be readily explained: the SF films are intrinsically rich in active binding sites, i.e., protonated amino for anionic dyes caused by formic acid added to promote the self‐assembly process.

We also fit the adsorption kinetics into both the pseudo first‐order and pseudo second‐order models (Figure 3F). Our results suggest that the adsorption data of five dyes fit well with the pseudo second‐order model (see Figure S4F, Supporting Information), suggesting that the rate‐limiting step in the dyes adsorption process is dominated by chemical adsorption rather than simple physical diffusion.^[^ ^58^ ^]^ The chemical adsorption prevents the dye molecules from escaping from the SF films, avoiding the secondary pollution.

The wastewater typically has a fluctuating pH value. Therefore, it is worthwhile to investigate the dependence of the adsorption capability of SF films on pH values. Here, we chose six different pH values (pH = 2, 3, 5, 7, 10, 12). Figure 3G suggests that the removal efficiency depends on the pH value. In particularly, the removal efficiency for anionic dyes such as RB4, DB2, ARS, and PB remains high for pH values ranging from 2 to 5. But it decreases significantly when the pH value is equal to 5 and it is stable again for pH from 7 to 12. In contrast, the removal efficiency for cationic dye RB increases slightly with an increase of the pH value. The dependence on the pH value can be attributed to the ionic interactions between SF films and dye molecules. Finally, the SF films do not degrade in both strong acid and alkaline environments, ideal for wastewater treatment.

**Figure 4:**
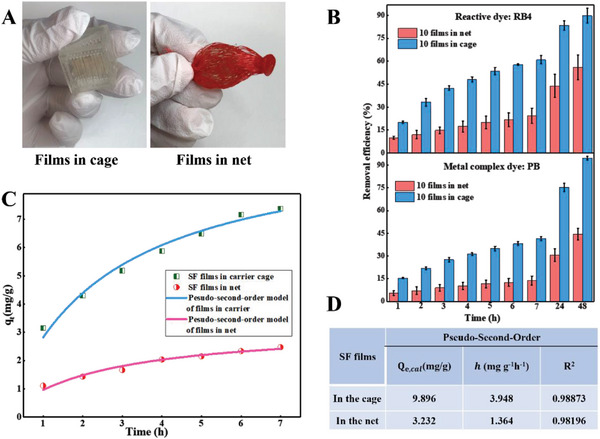
Dye removal using SF films in a carrier cage and a net, respectively. A) SF films in the 3D‐printed carrier cage and net, respectively. B) Reactive dye RB4 and metal complex dye PB removal efficiency using ten films with the size of 1.25 cm × 1.25 cm in the carrier cage and the net. C) The RB4 adsorption kinetics models of the SF films in the 3D‐printed carrier cage and the net, respectively. D) The kinetic parameters for the adsorption of RB4 into SF film in the carrier cage and the net, respectively.

When assembling the SF films into the WMTS, densely packed films could block the active binding sites and lower the removal efficiency of wastewater treatment.^[^ ^59^ ^]^ Herein, we design a customized 3D‐printed carrier cage to hold the SF films, which is fixed onto the WMTS holder (Figure 1A,C). To compare the dye adsorption capability of the SF films inserted into the carrier cage with those densely packed, we inserted ten films to a carrier cage (as the experimental group) and to a net (as the control group), respectively (**Figure** **4**A). SF films in the customized carrier cage show a higher and more stable removal efficiency (at least 183% improvement) for both RB4 and PB compared with those densely packed in the net (Figure 4B and Figure S5, Supporting Information). To further explain the adsorption processes of SF films in the cage setting and in the net setting, the adsorption data were fitted into the pseudo‐second‐order kinetic model (see the Experimental Section for details) (Figure 4C,D and Figure S6, Supporting Information). Evidently, the adsorption capacity *Q*_e,cal_and the initial adsorption rate *h* of the SF films in the cage (equation in the Experimental Section) are three times higher than those of the SF films in the net, suggesting that the customized carrier cage does not block the active binding sites of the SF films.

### 2.4 The WMTS Performance and Distribution

**Figure 5:**
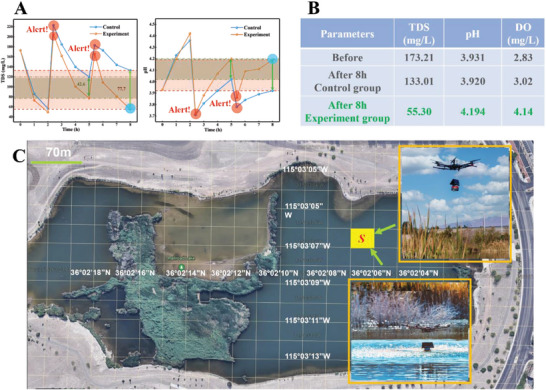
WMTS performance and distribution. A) The comparison of the water quality TDS and pH values during the treatment process with twice abnormal alters for experiment group using WMTS and control group; B) the water quality indices (TDS, pH, and DO) before and after the treatment with and without WMTS. C) The distribution and delivery of WMTS based on the grid division of the natural water body. The insets show the WMTS delivered by the UAVs.

To demonstrate the adaptability of WMTS, we deployed it into an artificial textile dyeing wastewater body (1 L) (reactive dye RB4) in the lab. The water quality indices, i.e., pH, TDS, and DO were monitored by the WMTS continuously. In comparison, the water quality indices of the control group were measured by the lab‐based sensors every hour manually. Initially, the purification strategy such as the number of SF films was set to the same for both situations (three cages with 15 SF films). During the treatment process, we intentionally added the same amount of dye in both cases twice to simulate a sudden increase of dye pollution. In both times, the WMTS successfully alerted in the GUI based on the ML‐based analysis results. Accordingly, the remedy strategy was modified based on the alert. Specifically, we add one more cage with five films at 2.4 h and another cage with four films at 5.2 h. In contrast, the purification strategy remained the same for the situation without adaptive WMTS. As a result, the water quality indices, i.e., TDS, pH, and DO improve significantly with adaptive WMTS after 8 h (**Figure** **5**A,B), compared to those without adaptive WMTS, suggesting that our smart adaptive water treatment can effectively remove the water pollution.

For practical applications in a larger water body, we can grid the natural water body and determine the necessary number of WMTS units for efficient treatment. Considering various control factors such as the effective sensing area of sensors, the maximum purification capability, and the wastewater pollution levels, the maximum purification volume for one WMTS unit (L) can be expressed as

(1) $$V=\frac{NmM}{C}$$

where *C* is the pollutant concentration (mg L^−1^ or ppm); *N* is the number of purification carriers to be used; *m* is the weight of adsorbents in one carrier (g); *M* is the maximum capacity of the adsorbents (mg g^−1^).

For a given water body with a volume of *V*_water_, the number of WMTS units (*n*) to be deployed is

(2) $$n=\frac{V_{\mathrm{water}}}{V}$$

For a highly polluted wastewater (high *C*) or a larger water body, we can either increase the number of WMTS units *n* or the carrier number *N* to achieve the required remediation.

Since we are not able to find a polluted natural water body for testing, here we can only provide a theoretical estimate. The maximum capacity *M* of the SF film used here for wastewater purification is 185 mg g^−1^ for reactive dye RB4, the weight of SF films in a fully loaded carrier *m* is 1 g, and the default carrier number *N* is 500. Assuming that a polluted water body has a volume of 2000 L and the average pollutant concentration is 100 ppm, the grid volume *V* and the number of WMTS units *n* can be calculated by Equations (1) and (2): 925 L and 2.

Next, we can map the grids into corresponding GPS coordinates, which can guide UAV to deliver the WMTS from the loading hub to the targeted grid area and WMTS is floated on the water. Figure 5C shows that for a demo, we dispatched a UAV to deliver the WMTS to the corresponding grid (inserts in Figure 5C and Figure S7, Supporting Information). In addition, the UAVs can also be dispatched to replace or recycle the WMTS units. Moreover, the UAVs can be preprogrammed with a specific GPS position of far‐flung and hard‐to‐reach watercourse and are versatile enough to access the desired depth.^[^ ^60^, ^61^, ^62^ ^]^ The UAV auto delivery system enables the WMTS to monitor water quality onsite and send data remotely with enhanced worker safety and operational efficiency during the whole procedure. The UAVs revolutionize the process of remote water sample collection and monitoring by protecting the operators from directly accessing to harsh environments.

## 3 Conclusion

In this article, we have successfully developed a highly integrated WMTS for efficient and sustainable treatment of natural water sources. The system consists of two functional components: the IoT‐based real‐time and continuous water monitoring unit and the bioadsorbent SF film‐based sustainable water purification unit. The system is integrated into a customized 3D‐printed holder and is solar‐driven for reliable and scalable long‐term and continuous natural water treatment applications. Specifically, the SF films are inserted into a 3D‐printed carrier cage to maximize the adsorption performance. For the adaptive water pollution treatment, the monitoring unit continuously collects the real‐time water quality data (i.e., DO, TDS and pH) and synchronizes the data to the Google cloud and remote terminal by the wireless communication. Simultaneously, the ML‐based cloud server automatically analyzes the uploaded data to assess the water quality in real‐time and assists in designing the water purification strategy with the optimal treatment efficiency. For practical applications in a large natural water body, the required number of WMTS units is determined using the defined grid rules and the required WMTS units can be delivered to the whole monitored water body (including the hard‐to‐reach sites) by UAVs according to the GPS coordinates of mapped grids. As a result, the whole water body can be adaptively and effectively treated with automatic operation and minimum maintenance. In summary, we provide a closed‐loop solution using the WMTS for the efficient and stable treatment of polluted natural water sources.

The main contribution of this work lies in the system‐level integration of state‐of‐the‐art ML techniques, a green and sustainable silk‐based bioadsorbent, and wireless IoT sensors for automated air‐delivery solar‐driven on‐site water monitoring and treatment. By real‐time capturing and analyzing water quality data, an optimized treatment strategy can be promptly implemented to improve the water quality. In addition, our formic acid‐based SF absorbents are different from the existing SF absorbents in literature. They can meet the stringent textile (dye) wastewater requirements (pH 2–12) and demonstrate high adsorption capacities toward all types of water‐soluble textile dyes.

Finally, in this article, we used the ML that can provide more accurate and timely information for water quality monitoring. The ML has the potential to improve the accuracy and efficiency of the monitoring process. The ML model can learn from historical data, identify patterns, and predict the expected range of water quality parameters for future years. This approach allows the system to anticipate changes in water quality and take necessary measures to address any potential risks before they become a problem. Furthermore, the model can continuously refine its predictions based on new data, enabling it to adapt to changes in water quality.

## 4 Experimental Section

### Water Monitoring System Design

In the monitoring system, the pH, TDS, and DO sensors purchased from Altas Scientific (Long Island, NY, USA) were used for collecting water quality data. The RPi 3B+ was used as a pocket controller for data processing as well as providing good internet connectivity. It operates on a 5 V, 400–690 mA (i.e., 2–3.5 W) power rating, which is much more energy and cost efficient than an actual computer. All sensors were connected to the RPi through a Tentacle T3 board to protect sensors from interfering with each other as well as eliminating external electrical noise. The system drained about 2 W at idle, and around 3.5 W while with a full workload under a Lightweight X11 Desktop Environment (LXDE). The whole system was powered by a hybrid system integrating a 22 W solar panel with a standard Pijuice Li‐ion 12 000 mAh battery. The typical power rate of the current WMTS was around 2.6 W. Therefore, the Pijuice battery could support the WMTS for 26 h. Even at the highest consumption, it could still work on battery alone for almost 18 h. The commercial solar cell panel and Pijuice Li‐ion battery could work in low temperature or rainy weather. In addition, the Pijuice GUI software could be installed on the RPi for power management. Meanwhile, the battery was charged by a solar panel (22 W, 5 V) during daylight for usage at night. The monitoring program was coded and implemented using Python run on the RPi core controller in order to send the measurements detected from sensors to the Google cloud. The GUI was designed upon PyQt. In addition, the pH sensor was calibrated by the low‐mid (pH 0–7) and mid‐high (pH 7–14) correction method. The TDS sensor was calibrated by zero air correction and the DO sensor was calibrated by zero solution correction.

### ML‐Based Real‐Time Analysis and Abnormality Detection

The Google cloud employed a one‐class SVM model (i.e., svm.OneClassSVM from the scikit‐learn package in Python) to distinguish between “normal” water quality data and “abnormal” data. The model utilized the radial basis function (RBF) as its kernel and took *nu =* 0.01 and *gamma =* 0.01 as its parameters to limit the training errors within 1%. The model was pre‐trained with practical data collected at Cornerstone Park in Henderson, Nevada, and created a range of “normal values.” Any data that fall outside of the “normal range” were considered as “abnormalities.” Once the real‐time quality data were uploaded to the Google cloud, it would be read via Google Sheets application programming interface (API) into the pre‐trained SVM model to be determined whether it belonged to “normal” or “abnormal” (i.e., within the “normal range” or not). With that, “abnormal” water quality could be detected and reported in real‐time, and such records could assist researchers to further develop a rapid treatment strategy for early detection and prevention of the abnormal changes of water quality.

### Study of Soluble Synthetic Dyes Removal Efficiency by SF Films

The removal capacity of SF films was determined by a batch of the adsorption experiments using five types of synthetic dyes at different concentrations. The dye adsorption experiments were performed by immersing SF films (1.25 cm × 1.25 cm) into 2.5 mL of dye aqueous solutions. Each residual dye concentration at the designated time point was determined by monitoring the absorbance value at their individual maximum adsorption wavelength using the UV‐vis spectroscopy with a 1 mL quartz microcell. Similar experimental procedures were also carried out to explore the influence of the solution pH values (2–12) on the removal efficiency. The initial pH of dye solution was adjusted to a given value by HCl or NaOH solution, accordingly. In addition, the heavy metal ions (Hg^2+^, Cr^6+^) adsorption experiments were conducted by immersing SF films (1.25cm × 1.25cm) into 2.5 mL of heavy metal ions aqueous solutions. The remained concentrations after the adsorption were determined by the inductively coupled plasma mass spectroscopy. The removal efficiency (%) and adsorption capacity or dye uptake *Q*_t_ and *Q*_e_ (mg g^−1^) were calculated from the following equations, respectively

(3) $$\mathrm{Removal}/\mathrm{adsorption}\mathrm{efficency}\left(\%\right)=\frac{C_{0}-C}{C_{0}}$$

(4) $$Q_{t}(\mathrm{mg}g^{-1})=\frac{C_{0}-C_{t}}{m}\times V$$

and

(5) $$Q_{e}(\mathrm{mg}g^{-1})=\frac{C_{0}-C_{e}}{m}\times V.$$

In the above, *Q*_t_(mgg^−1^) and *Q*_e_(mgg^−1^) are the adsorption capacities of dyes at time *t* (hour) and equilibrium. *C*_0_ is the initial dye concentration (ppm or mg L^−1^); *C*_t_ is the dye concentration after adsorption for *t* hours (ppm or mg L^−1^); *V* (L) is the volume of the dye solution and *m* (g) is the weight of the SF film. The equilibrium dye adsorption capacity *Q*_e_ is expressed in mg dye g^−1^ SF film.

### Study of SF Films in the Carrier Cage for Dye Removal Efficiency

The SF films (1.25 cm × 1.25 cm) were put into the customized 3D printing cage to determine the dye removal efficiency. The same number of SF films (1.25 cm × 1.25 cm) was also put into the net and then tightly wrapped. The removal efficiency was determined by putting the cage with SF films and net with SF films into the dye solution (reactive dye RB4 and metal complex dye PB), respectively. At the designated time points, the concentration of the dye solution was determined by monitoring the absorbance value at their individual maximum adsorption wavelength using the UV‐vis spectroscopy.

### Study of the WMTS Adaptive Treatment Performance

The RB4 solution 500 ppm was prepared with two groups (one for experiment group and the other for control group). During the treatment process, the pH and TDS values of the experimental group were tested in real‐time and automatically saved every hour by the WMTS, while those of the control group were determined manually in the lab. Since the DO value was primarily determined by the biochemical activity in the water, only the DO value was tested before and after the treatment for assessment. Two time points were picked during the treatment process and the RB4 concentrations of both groups were increased to simulate sudden pollution events. Once the alerts were received in the UI, those were responded by changing the number of SF films in usage.

## Conflict of Interest

The authors declare no conflict of interest.

## Supporting information

Supporting Information

## Data Availability

The data that support the findings of this study are available from the corresponding author upon reasonable request.
