# Supplementary material for: Automated Drone‐Delivery Solar‐Driven Onsite Wastewater Smart Monitoring and Treatment System
Source: Adv Sci (Weinh). 2023 Jun 26;10(24):2302935. doi: 10.1002/advs.202302935 (PMC10460888; doi:10.1002/advs.202302935)
Supplement: Supplementary file 1 — Supporting Information [file ADVS-10-2302935-s001.pdf]

ADVANCED SCIENCE
Open Access

Supporting Information

for *Adv. Sci.*, DOI 10.1002/advs.202302935

Automated Drone-Delivery Solar-Driven Onsite Wastewater Smart Monitoring and Treatment System

Fengjie He, Ming Zhu, Jiawei Fan, Edwin Ma, Shengjie Zhai and Hui Zhao**

Supplementary Materials for

Automated drone-delivery solar-driven onsite wastewater smart monitoring and treatment system

Fengjie He[1], Ming Zhu[2], Jiawei Fan[2], Edwin Ma[3], Shengjie Zhai[2*], and Hui Zhao[1*]

[1]Department of Mechanical Engineering, University of Nevada, Las Vegas, NV, USA, 89154
[2]Department of Electrical and Computer Engineering, Engineering, University of Nevada, Las Vegas, NV, USA, 89154
[3]Ed W. Clark High School, Las Vegas, NV, USA, 89102
*Corresponding author. Email: hui.zhao@unlv.edu; shengjie.zhai@unlv.edu

This PDF file includes:

Supplementary Text
Figures S1 to S7
Tables S1 to S2
References

Supplementary Text

Section S1. Materials

Bombyx mori cocoons were obtained from Southwest University (Chongqing, China). Pricion blue H-5R (PB), Rhodamine B(RB), Direct blue 2 (DB2), Alizarin Red S (ARS), Reactive blue 4 (RB4), formic acid, sodium carbonate (Na_2CO_3), lithium bromide, sodium hydroxide (NaOH) and hydrochloric acid (HCl) were purchased from VWR (Atlanta, GA, USA). Hexavalent Chromium (Cr^{6+}) and mercury (Hg) standard solution were purchased from High-Purity Standards (North Charleston, SC, USA). All solutions were prepared with ultra-pure (UP) water. All the chemicals were used directly without further purification.

Section S2. Implementation of the water monitoring system

To test the water monitoring system performance, we set the assembled monitoring system to the Railroad Lake of Cornerstone Park in Henderson, Nevada (Latitude: 36°2'7.692" N Longitude: 115° 3' 19.7748" W). Later, we run the automatic mode in the UI to read and save the DO, pH, and TDS values every hour from 12:00 pm to 7:00 pm.

Section S3. Preparation and characterization of SF silk fibroin films

The *Bombyx mori* cocoons were cut into dime size pieces to boil in 0.02M Na_2CO_3 solution for one hour to fully degum the sericin. Then the degummed silk fibers were rinsed with UP water and dried overnight at room temperature (20°C). The dried silk fibers were then dissolved in 9.3 M LiBr solution at 60 °C for four hours. The obtained solution was slowly dripped into ethanol and precipitated out into small firoin precipitants. These SF precipitants were then rinsed several times with UP water to remove all the ethanol and LiBr residuals and were dried overnight in the oven. The dried SF was finally dissolved in the 98% formic acid to form homogenous SF formic acid solution. SF films were fabricated by casting the SF formic acid solution onto a petri dish and evaporating at room temperature (20°C) for overnight.

The molecular structure of SF films was analyzed by the attenuated total reflectance-Fourier transform infrared (FTIR-ATR) spectrometer (BIO-RAD FTS-7000) using a frequency of 2.5kHz, a filter setting of 1.2, and a UDR setting of 2. The film was scanned 64 times at a resolution of 8cm^{-1} over a wavelength of 400-4000cm^{-1}. To get each secondary structure content of SF films, the SF film secondary structure was analyzed by FTIR-ATR and deconvolved by the software PeakFit to quantify the secondary structure content of amide I region from 1600 to 1703cm^{-1} (Supplementary Table 2).

Section S4. Adsorption isotherm and kinetics

To estimate the SF film's adsorption process and capacities of different dyes, the experimental equilibrium data at room temperature were fitted with two well-known isotherm models (Langmuir and Freundlich isotherm model).

The Langmuir isotherm model assumes that the adsorption occurs at a specific homogeneous adsorbent surface with a finite number of adsorption sites, and is generally applied in the monolayer adsorption process(*1*, *2*). The Langmuir isotherm can be expressed as,

$$\frac{1}{Q_e}=\frac{1}{Q_m}+\frac{1}{C_eQ_mK_L} \quad (1)$$

The Freundlich isotherm models assumes that the adsorption occurs at a heterogeneous adsorbent surface with multilayer, which can be expressed as(*3*),

$$\ln Q_e=\ln K_F+\frac{1}{n}\ln C_e . \quad (2)$$

In the abo

constant, K_F ((mg g^{-1}) (L mg^{-1}) ^{n}) is the Freundlich model; and n is the Freundlich constant correlated to the adsorption intensity.

To further investigate the adsorption kinetics and mechanism of dyes into the SF films, the pseudo-first-order model and pseudo-second-order model were used to fit the kinetic data. The two models can be expressed as the following equations, respectively(*2*, *4*):

$$\ln(Q_e-Q_t)=\ln Q_e-k_1t \quad (3)$$

$$\frac{t}{Q_t}=\frac{1}{k_2Q_e^2}+\frac{t}{Q_e} \quad (4)$$

$$h=k_2Q_e^2 , \quad (5)$$

where Q_e (mg/g) and Q_t (mg/g) are the respective number of dyes adsorbed per unit weight of SF films at equilibrium and at time t (hour), and k_1 (h^{-1}) and k_2 (g mg $^{-1}$ h^{-1}) are the respective rate constants for the pseudo-first-order model and pseudo-second-order model. Furthermore, the h (mg g $^{-1}$ h^{-1}) is the adsorption rate of the adsorption process obtained from the pseudo-second-order model(*5*).

Section S4. Fabrication of carriers and holder using 3D printing

We used the SOLIDWORKS 2019 software to design the carrier for SF films and the holder for the water monitoring and treatment system. Then, the file from the software was exported into an STL file format that was sent to the 3D printer for printing (Miicraft Ultra).

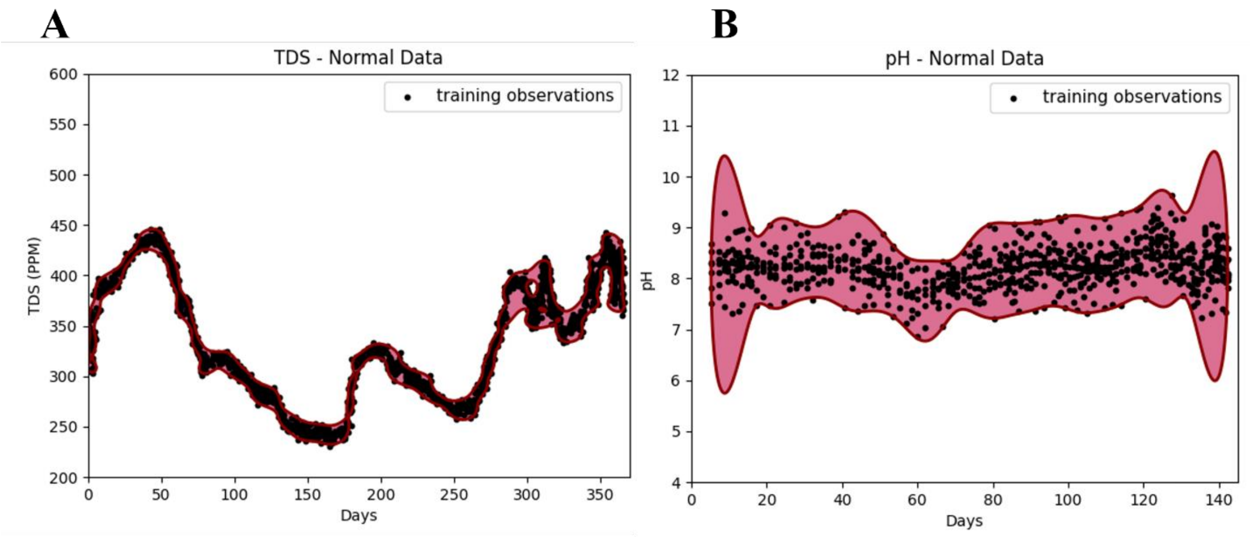

Figure S1. The machine learning results. (A) The training outcomes of the SVM models for TDS indices, and (B) The training outcomes of the SVM models for the pH indices.

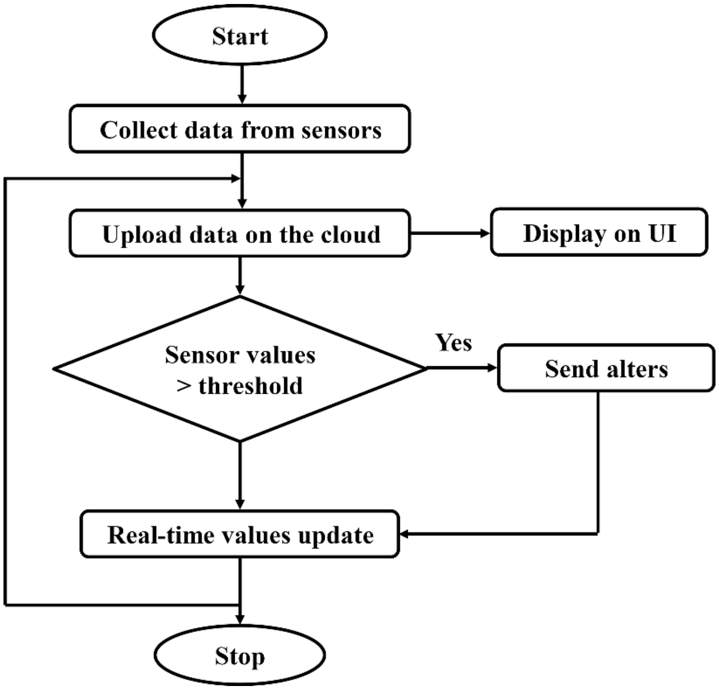

Figure S2. The workflow of the monitoring system of the WMTS.

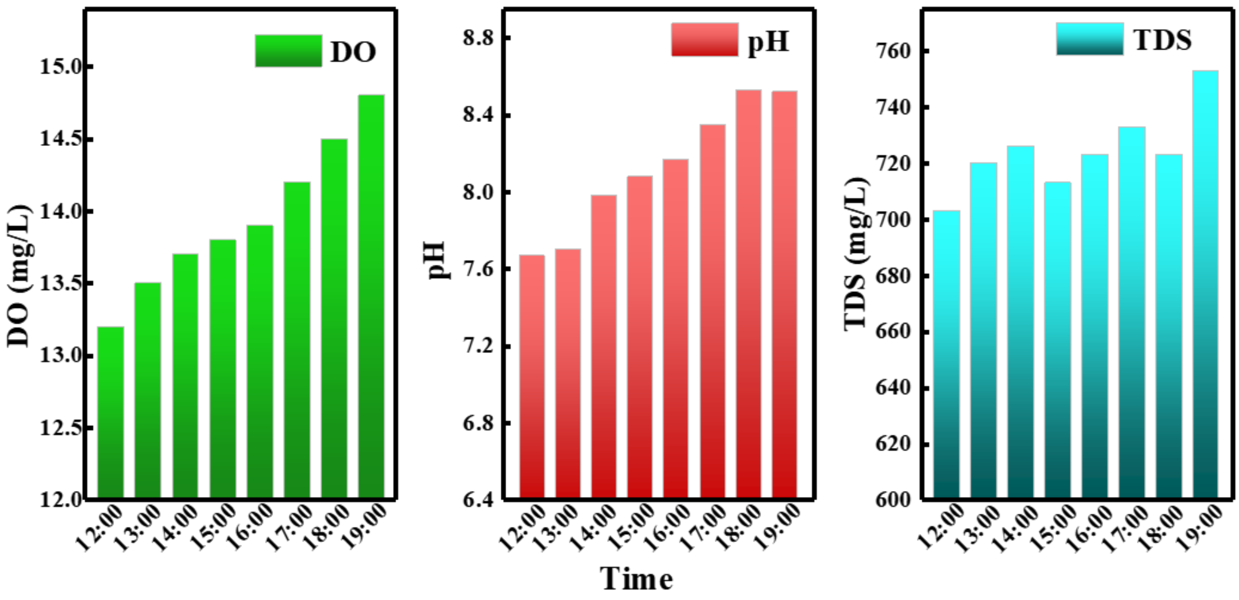

Figure. S3. The monitoring results of the WMTS in the cold and rainy day.

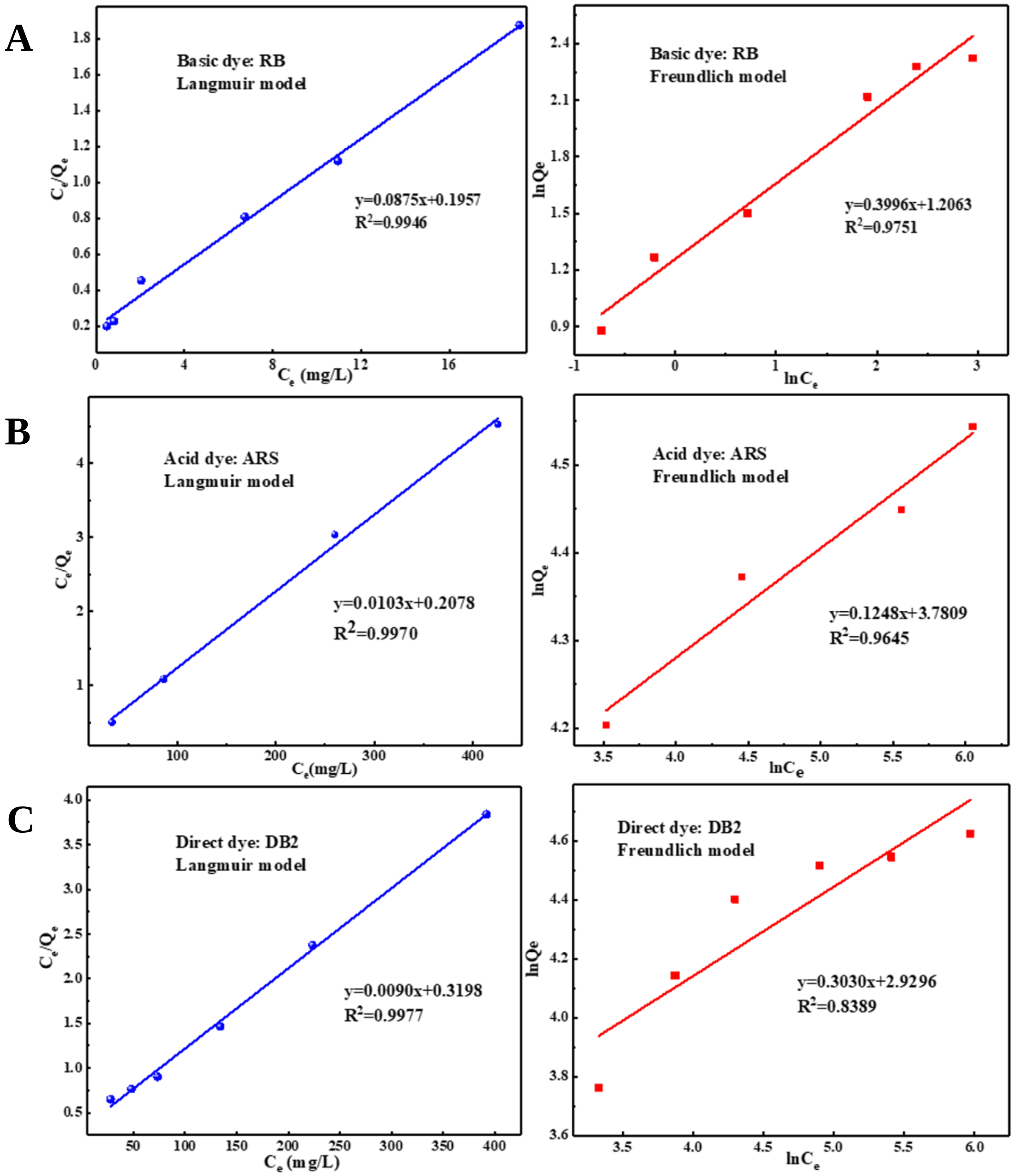
A
Basic dye: RB
Langmuir model
y=0.0875x+0.1957
R2=0.9946
Ce/Qe
Ce (mg/L)
Basic dye: RB
Freundlich model
y=0.3996x+1.2063
R2=0.9751
lnQe
lnCe
B
Acid dye: ARS
Langmuir model
y=0.0103x+0.2078
R2=0.9970
Ce/Qe
Ce(mg/L)
Acid dye: ARS
Freundlich model
y=0.1248x+3.7809
R2=0.9645
lnQe
lnCe
C
Direct dye: DB2
Langmuir model
y=0.0090x+0.3198
R2=0.9977
Ce/Qe
Ce (mg/L)
Direct dye: DB2
Freundlich model
y=0.3030x+2.9296
R2=0.8389
lnQe
lnCe

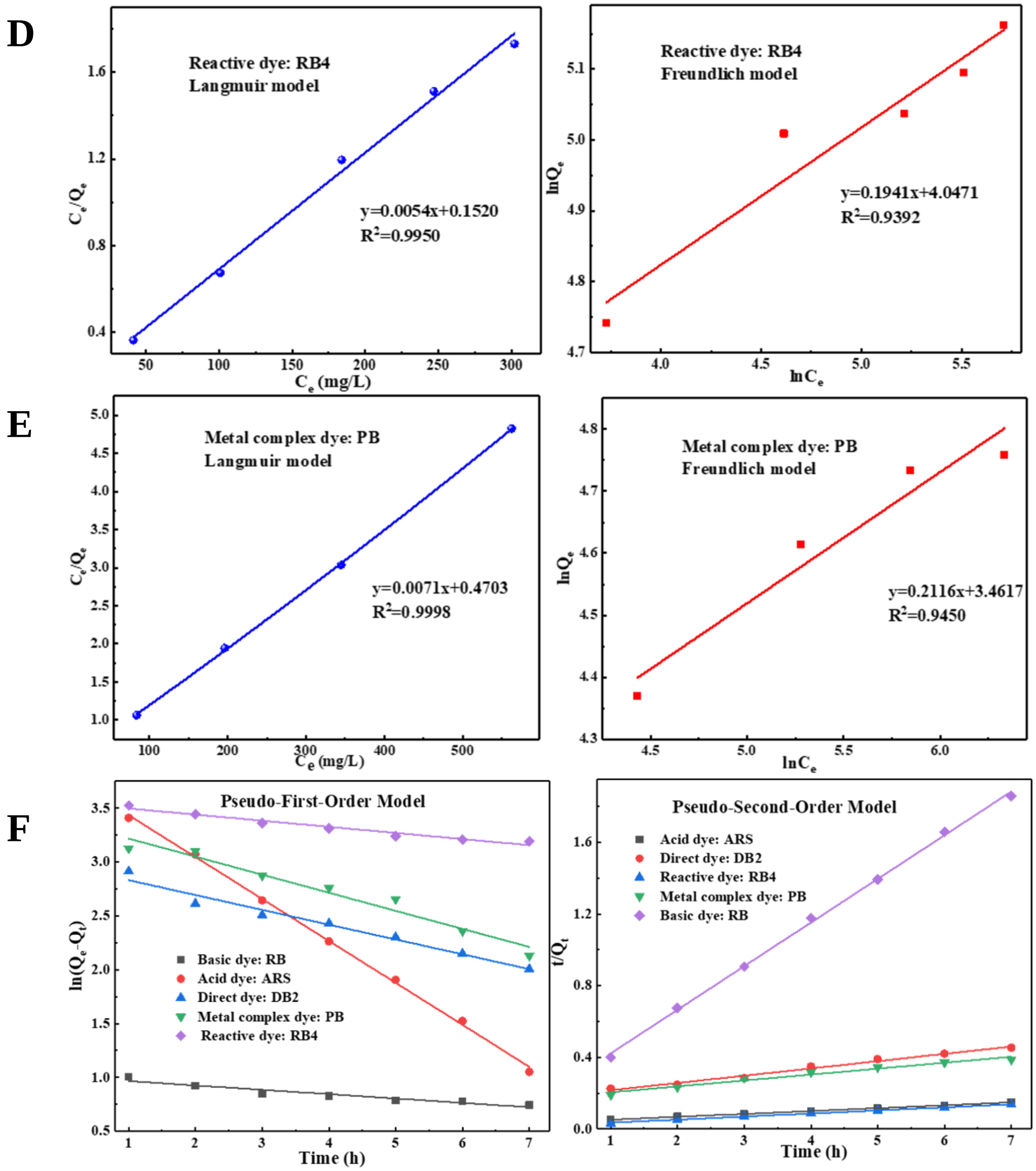

Figure S4. Adsorption isotherms and kinetic. A-E, Linear plots of Langmuir (left), Freundlich (right) isotherms for adsorption of five dyes on SF films, respectively. F, Linear plots of the Pseudo-Second-Order kinetic for dyes adsorption on SF films.

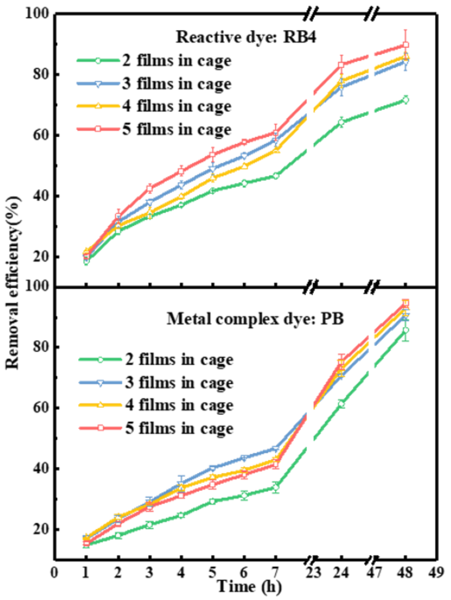

Figure S5. Dye removal efficiency using SF films in cage and net, respectively. Reactive dye RB4 removal efficiency using 2-5 SF films (size of 1.25cm×1.25cm) in cage, respectively (top). Metal complex dye PB removal efficiency using 2-5 SF films (size of 1.5cm×1.5cm) in cage, respectively (bottom).

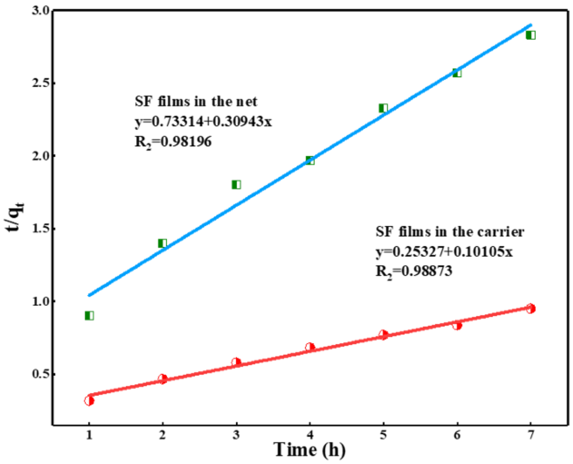

Figure S6. Linear fitting of Pseudo-second-order of the SF films in the carrier and net, respectively.

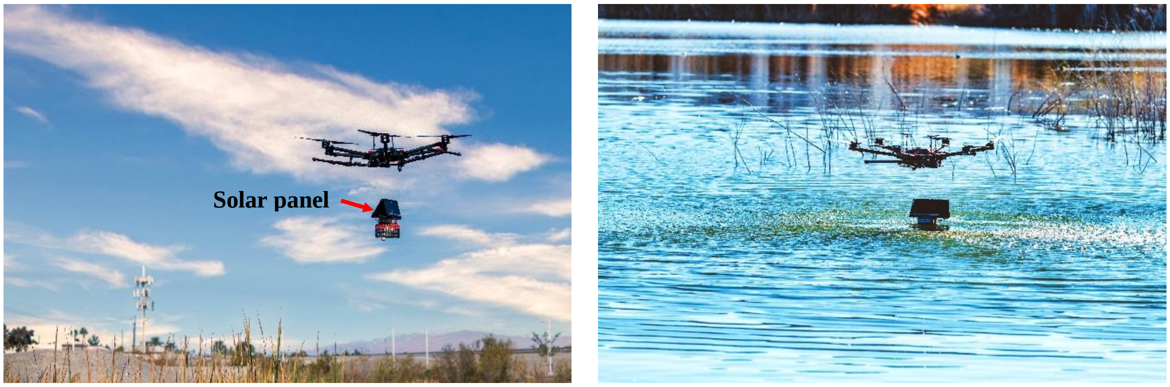

Figure S7. The WMTS delivered by the Unmanned Aerial Vehicles (UAVs).

Silk secondary structure	Water-based (% content)	Formic acid-based (% content)
Side chains	10.5	12.2
β-sheet	27.5	42.2
Random coils	40.6	25.1
α-helix	9.7	8.4
Turns	11.7	12.1

Table S1. Silk Secondary Content for Water-based and Formic Acid-based Film Surface.

Wavenumber range (cm^{-1})	Secondary structure assignment
1605-1615	Side chains
1616-1637 and 1697-1703	β-sheet
1638-1655	Random coils
1656-1662	α-helix
1663-1696	Turns

Table S2. Vibrational band assignments for the amide I region of silk fibroin(*6*).

References

1. I. Langmuir, The adsorption of gases on plane surfaces of glass, mica and platinum. *J. Am. Chem. Soc.* **40**, 1361–1403 (1918).
2. X. X. Zhou, Y. J. Li, J. F. Liu, Highly Efficient Removal of Silver-Containing Nanoparticles in Waters by Aged Iron Oxide Magnetic Particles. *ACS Sustain. Chem. Eng.* **5**, 5468–5476 (2017).
3. R. Mallampati, L. Xuanjun, A. Adin, S. Valiyaveettil, Fruit peels as efficient renewable adsorbents for removal of dissolved heavy metals and dyes from water. *ACS Sustain. Chem. Eng.* **3**, 1117–1124 (2015).
4. Y. S. Ho, G. McKay, Kinetic models for the sorption of dye from aqueous solution by wood. *Process Saf. Environ. Prot.* **76**, 183–191 (1998).
5. Y. Zhao, W. Li, Z. Liu, J. Liu, L. Zhu, X. Liu, K. Huang, Renewable Tb/Eu-Loaded Garlic Peels for Enhanced Adsorption of Enrofloxacin: Kinetics, Isotherms, Thermodynamics, and Mechanism. *ACS Sustain. Chem. Eng.* **6**, 15264–15272 (2018).
6. B. D. Lawrence, F. Omenetto, K. Chui, D. L. Kaplan, Processing methods to control silk fibroin film biomaterial features. *J. Mater. Sci.* **43**, 6967–6985 (2008).
